# Vanishing large ovarian cyst with thyroxine therapy

**DOI:** 10.1530/EDM-13-0050

**Published:** 2013-11-01

**Authors:** Pramila Dharmshaktu, Aditya Kutiyal, Dinesh Dhanwal

**Affiliations:** 1 Department of MedicineMaulana Azad Medical CollegeNew DelhiIndia

## Abstract

A 21-year-old female patient recently diagnosed with severe hypothyroidism was found to have a large ovarian cyst. In view of the large ovarian cyst, she was advised to undergo elective laparotomy in the gynaecology department. She was further evaluated in our medical out-patient department (OPD), and elective surgery was withheld. She was started on thyroxine replacement therapy, and within a period of 4 months, the size of the cyst regressed significantly, thereby improving the condition of the patient significantly. This case report highlights the rare and often missed association between hypothyroidism and ovarian cysts. Although very rare, profound hypothyroidism that can cause ovarian cysts in an adult should always be kept in the differential diagnosis to avoid unnecessary ovarian surgery.

**Learning points:**

- Hypothyroidism should be considered in the differential diagnosis of adult females presenting with multicystic ovarian tumours.
- Adequate thyroid hormone replacement therapy can prevent these patients from undergoing unnecessary and catastrophic ovarian resection.
- Surgical excision should be considered only when adequate thyroid replacement therapy fails to resolve ovarian enlargement.
- In younger women with ovarian cysts, it is also desirable to avoid unnecessary surgery so as to not compromise fertility in the future.

## Background

Ovarian cysts are a common cause for gynaecological surgery. However, some ovarian cysts arise due to endocrine disorders and hence do not require any surgical intervention. Primary hypothyroidism is a common endocrine abnormality resulting from thyroid hormone deficiency that in turn may lead to multiple-system impairment.

## Case presentation

A 21-year-old unmarried female patient presented to our gynaecology department with complaints of increased blood loss during menstruation with passage of blood clots for the past 4 months. She had swelling in hands and feet with facial puffiness. She also had easy fatigability, shortness of breath on moderate exertion and lower abdominal pain for the past 2 months. The patient also complained of excessive dryness of skin, intolerance to cold, weight gain, heaviness of voice and increased hair fall. On physical examination, she was found to have facial puffiness with the presence of mild swelling in the feet. Her weight was 72 kg and her skin was dry. Deep tendon reflexes were prolonged on examination. A clinical diagnosis of hypothyroidism was made, and the patient was investigated further.

## Investigation

**Figure 1:**
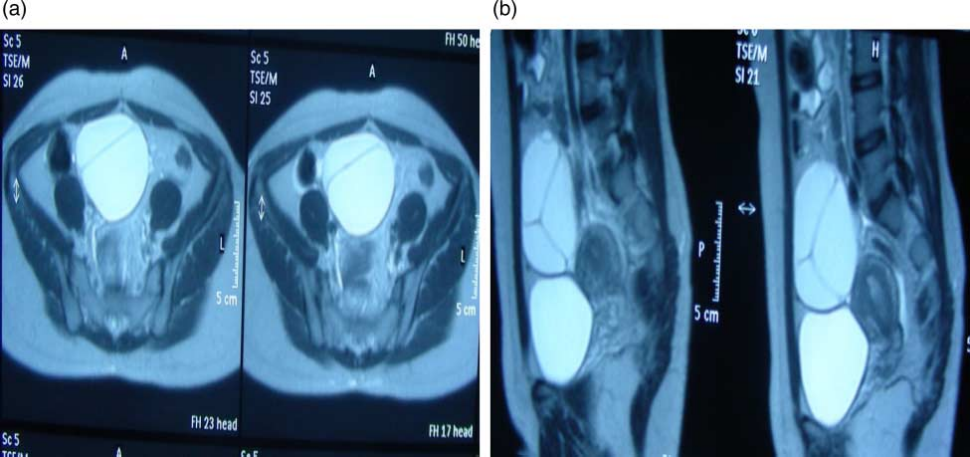
MRI scan of the pelvis showing a large multiseptated lobulated cystic lesion arising from the right ovary: (a) axial section view and (b) sagittal section view.

The patient was admitted to our clinic, and an ultrasound scan of the pelvis region was performed, which revealed a multicystic lesion in the pouch of Douglas. The magnetic resonance imaging (MRI) scan of pelvis revealed a large multiseptated and lobulated cystic lesion, measuring 8×8×6 cm, arising from the right ovary (Fig. 1a and b). CA-125 levels were within the normal range, and no abnormality was detected on performing an ultrasound scan of the thyroid gland. The patient had gone to some other hospital with similar complaints and was advised to undergo a thyroid function test, which revealed thyroid-stimulating hormone (TSH) levels above 100.0 μIU/ml (normal range: 0.34–4.25) and free thyroxine (T_4_) levels=0.70 ng/l (normal range: 0.8–1.8 ng/l). As she was not on any treatment, she was prescribed T_4_ tablet (100 μg/day) in our clinic after the detection of the ovarian cyst. Other haematological and biochemical profiles of the patient were within the normal range.

## Treatment

The patient was started on oral tablet of l-T_4_ at a dose of 100 μg/day.

## Outcome and follow-up

**Figure 2:**
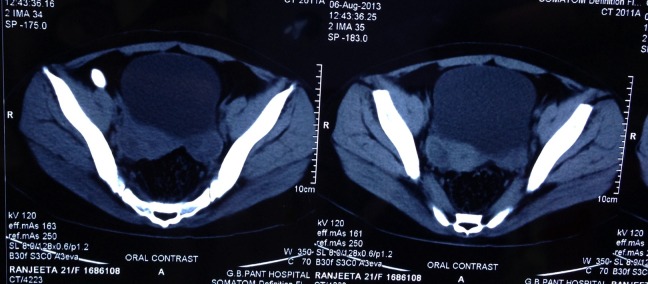
CT scan of the pelvis showing normal ovaries with the presence of only follicular cysts after 6 months of therapy.

In view of the large ovarian cyst, the patient was advised to undergo elective laparotomy, and on routine preanaesthetic evaluation, a mandatory opinion of an endocrinologist was taken for hypothyroidism. Surgery was withheld, and only T_4_ therapy was advised in our clinic. Review ultrasound scan of the pelvis region was performed 4 months later, and it revealed a 4×1.4 cm-sized cystic lesion with few thin septations within the cyst in the right ovary. The patient responded well to conservative management, and a significant regression in the size of the cystic lesion was observed at the end of the 4-month follow-up, and complete resolution was observed after 6 months without any need for surgical intervention (Fig. 2).

## Discussion

Ovarian function, i.e. production of steroid hormones and ova, is subject to regulation by endocrine factors derived from the brain. This brain–gonadal axis is the core unit for the maintenance of endocrine balance and fertility. Hypothyroidism may cause reproductive disorders as well. Occasionally, concomitant ovarian cyst formation is reported as the Van Wyk and Grumbach syndrome (1) in juvenile primary hypothyroidism. It is less commonly seen in adults. Failure to recognize hypothyroidism as an aetiology of ovarian cysts could lead to inadvertent oophorectomy.

Ovarian hyperstimulation is a condition that can result from reproductive endocrine dysfunction and may resolve without surgery only after endocrine correction. Usually, ovarian hyperstimulation syndrome (OHSS) is caused by iatrogenic superovulation. Excessive amounts of exogenous follicle-stimulating hormone (FSH) stimulate multiple follicular growth simultaneously. In some rare cases, spontaneous OHSS related to pregnancy has been described to be dependent on the activating mutations of the FSH receptor (*FSHR*) gene, causing ovarian hyper-responsiveness to circulating FSH or even cross-responsiveness of FSHR to hormones having a structure similar to FSH, such as human chorionic gonadotrophin or TSH (2) (3). Ectopic gonadotrophin adenoma secreting FSH can also present with multiple follicular cysts in ovaries (4) (5). Without considering these endocrine disorders as a possible aetiology, clinicians are likely to assume a diagnosis of neoplasm, leading to unnecessary ovarian surgery.

Hypothyroidism is another endocrine disorder associated with ovarian hyperstimulation, yet it is often ignored during its evaluation. Spontaneous OHSS cases have been reported in pregnant women with hypothyroidism (6) (7) (8). Since Van Wyk & Grumbach had first described the combination of multicystic ovaries, juvenile hypothyroidism and precocious puberty in 1960, sporadic cases of this syndrome have been reported in prepubertal and adolescent girls (9) (10). However, very few cases have been reported in adults aged 19–26 years (11) (12).

Ovarian cysts are common in postmenopausal women, although the prevalence is lower than that in premenopausal women. These ovarian cysts do not always require treatment. In premenopausal women, simple ovarian cysts often resolve on their own within 1–2 months without treatment, but large, multiloculated and painful or symptomatic cysts usually require treatment. Both ovarian enlargement and ovarian cysts are associated with hypothyroidism. A decrease in ovarian volume, resolution of ovarian cysts and reversal of the polycystic ovary syndrome-like appearance, together with improvement in serum hormone levels, has been shown to occur after the achievement of euthyroidism (11). Although polycystic ovaries are more commonly associated with primary hypothyroidism, our patient was found to have a large multiseptated ovarian cyst, and consistent regression of the ovarian cyst after thyroid hormone replacement therapy supports a causal relationship between hypothyroidism and ovarian stimulation in the present case.

The association of multicystic ovarian disease with hypothyroidism has been described in the literature (13) (14) (15). Various mechanisms have been postulated, which include altered oestrogen metabolism, hypothalamic–pituitary dysfunction and deranged prolactin metabolism. According to Anasti *et al*. (16), ovarian enlargement in severe hypothyroidism is probably due to the stimulation of FSHRs by unusually high TSH levels proven to have a weak FSH-like activity. It has been shown that TSH could interact directly with the FSHRs to elicit gonadal stimulation, because TSH has a small FSH- and luteinizing hormone (LH)-like effect.

Aghajanova *et al*. (17) discussed the distribution and activity of the TSH receptor (TSHR) and the thyroid hormone receptor α1 (TRα1), TRα2 and TRβ1 in human ovarian tissue and granulosa cells using immunohistochemistry, reverse-transcriptase PCR (RT-PCR), quantitative PCR and immunoassays. Strong immunostaining of TSHR, TRα1 and TRβ1 was demonstrated in the ovarian surface epithelium and in the oocytes of primordial, primary and secondary follicles, with minimal staining in the granulosa cells of secondary follicles, which supports the view that TSHR and TRs may participate in the regulation of ovarian function (17). The thickened endometrium in our patient was probably due to excessive amounts of TSH with amplification of FSH action and release by low LH thus leading to dysfunctional uterine bleeding and anaemia. The FSHR is expressed during the luteal phase in the secretory endometrium of the uterus. Marked clinical improvement was observed in the patient as menstrual cycles became regular, anaemia got corrected and abdominal pain got relieved. The regression of the ovarian cyst was observed following administration of thyroid hormone. Yamashita *et al*. (11) reported the regression of both pituitary and ovarian cysts after administration of thyroid hormone in a case of primary hypothyroidism. Our patient was having long-standing hypothyroidism, as indicated by the duration of her clinical features and very high serum TSH levels. It is also very rare to come across untreated patients with such high TSH levels. This case also highlights that such patients should be treated as early as possible to avoid complications.

There is evidence that supplementation with thyroid hormone can lead to the complete regression of multicystic ovarian cysts. Surgical exploration in these cases should be performed only in emergency cases such as ovarian torsion and rupture. Surgical excision should be considered only when adequate thyroid replacement therapy fails to resolve ovarian enlargement. Hypothyroidism and other endocrine disorders should be considered in the differential diagnosis of adult females presenting with multicystic ovarian tumours to avoid unnecessary ovarian resection and young patients with ovarian cysts should be recommended to undergo screening for hypothyroidism.

## Patient consent

Full informed consent was obtained from the patient before drafting the case report.

## Author contribution statement

Drs P Dharmshaktu and A Kutiyal were the treating resident doctors in the ward and mainly responsible for drafting this case report. Dr D Dhanwal provided expert opinion on the patient's diagnosis and further management.
